# Integrating the Ribonucleic Acid Sequencing Data From Various Studies for Exploring the Multiple Sclerosis-Related Long Noncoding Ribonucleic Acids and Their Functions

**DOI:** 10.3389/fgene.2019.01136

**Published:** 2019-11-12

**Authors:** Zhijie Han, Jiao Hua, Weiwei Xue, Feng Zhu

**Affiliations:** ^1^College of Pharmaceutical Sciences, Zhejiang University, Hangzhou, China; ^2^School of Pharmaceutical Sciences, Chongqing University, Chongqing, China; ^3^School of Mathematics, Harbin Institute of Technology, Harbin, China

**Keywords:** ribonucleic acid sequencing, multiple sclerosis, long non-coding ribonucleic acids, meta-analysis, function analysis

## Abstract

Multiple sclerosis (MS) is a chronic fatal central nervous system (CNS) disease involving in complex immunity dysfunction. Recently, long noncoding RNAs (lncRNAs) were discovered as the important regulatory factors for the pathogenesis of MS. However, these findings often cannot be repeated and confirmed by the subsequent studies. We considered that the small-scale samples or the heterogeneity among various tissues may result in the divergence of the results. Currently, RNA-seq has become a powerful approach to quantify the abundances of lncRNA transcripts. Therefore, we comprehensively collected the MS-related RNA-seq data from a variety of previous studies, and integrated these data using an expression-based meta-analysis to identify the differentially expressed lncRNA between MS patients and controls in whole samples and subgroups. Then, we performed the Jensen-Shannon (JS) divergence and cluster analysis to explore the heterogeneity and expression specificity among various tissues. Finally, we investigated the potential function of identified lncRNAs for MS using weighted gene co-expression network analysis (WGCNA) and gene set enrichment analysis (GSEA), and 5,420 MS-related lncRNAs specifically expressed in the brain tissue were identified. The subgroup analysis found a small heterogeneity of the lncRNA expression profiles between brain and blood tissues. The results of WGCNA and GSEA showed that a potential important function of lncRNAs in MS may be involved in the regulation of ribonucleoproteins and tumor necrosis factor cytokines receptors. In summary, this study provided a strategy to explore disease-related lncRNAs on genome-wide scale, and our findings will be benefit to improve the understanding of MS pathogenesis.

## Introduction

Multiple sclerosis (MS) is a chronic fatal neurodegenerative disease involving in complex immunity [central nervous system (CNS)] (Sospedra and Martin, 2005; Frohman et al., 2006; Li et al., 2018). Based on the 2014 statistics of the Atlas of MS investigation, the estimated number of the people afflicted with the MS worldwide has reached approximately 2.3 million (Browne et al., 2014). Although much remains unknown about the molecular etiology of MS, more and more studies showed that the dysregulation of transcriptional processes could potentially contribute to the pathogenesis of MS (Li et al., 2017; Selmaj et al., 2017; Angerer et al., 2018; Cheng et al., 2018; Han et al., 2018b; Zhang et al., 2019).

Recently, long noncoding RNA (lncRNA), one of the non-protein-coding genes whose transcripts are longer than 200 nucleotides, has been discovered as the important regulatory factor of immune system and pathogenesis of CNS disorders including MS (Gomez et al., 2013; Ng et al., 2013; Dong et al., 2015; Cheng et al., 2016; Santoro et al., 2016; Zhang et al., 2016; Chen et al., 2017; Eftekharian et al., 2017; He et al., 2017; Cheng et al., 2018; Yin et al., 2019). However, for MS, these results often cannot be repeated and confirmed by subsequent study. For example, multiple variants of the lncRNA antisense non-coding RNA in the INK4 locus (*ANRIL*) are found significantly associated with the risk of MS through the haplotype analysis of blood samples (Rezazadeh et al., 2018). But following study reveals that the function of *ANRIL* does not contribute the pathogenesis of MS in blood, cortex, and cerebellum tissues (Pahlevan Kakhki et al., 2018). Study showed a significant upregulation of lncRNA *MALAT1* in MS blood tissues (Cardamone et al., 2019), while the expression of *MALAT1* was found markedly decreased in MS brain by the subsequent study (Masoumi et al., 2019). Moreover, another study found that *MALAT1* is not significantly differentially expressed between MS patients and controls (Gharesouran et al., 2019). We considered that the small-scale samples or the heterogeneity among various tissues may result in the divergence of the results.

Currently, specifically for lncRNAs, using RNA-seq data to quantify abundance of the transcripts has become very powerful approach compared with the traditional ones (e.g., gene microarray) (Wang et al., 2009). Particularly, almost all of the expression of the known lncRNA transcripts can be measured using RNA-seq data, but this proportion is just approximately 0.1 to 10.6% by the method of probe re-annotation using various types of microarrays (Du et al., 2013; Fang et al., 2018; Yang et al., 2019). Moreover, lncRNA abundance quantification using RNA-seq data also shows higher accuracy based on its deep read coverage, while the re-annotation approach only requires the sequence match of 1 to 4 probes when quantifies lncRNA abundance (Du et al., 2013; Gellert et al., 2013; Li et al., 2019). A previous study reported that by paying attention to some aspect of library and sequencing process [i.e., poly-A tail selection, paired-end sequencing, and sequencing of double-stranded complementary DNA (cDNA)], the lncRNAs are more easily and more accurately identified through RNA-seq (Ilott and Ponting, 2013).

In this study, we thus selected all MS-related RNA-seq data in a variety of studies by searching three authoritative public databases: GEO DataSets (Barrett et al., 2013), EBI-EMBL ArrayExpress (Athar et al., 2019), and DDBJ Sequence Read Archive (Ogasawara et al., 2013) using the keyword “multiple sclerosis.” Then, we used these RNA-seq data to perform expression quantification of the lncRNA in each of the selected studies. Next, we integrated the lncRNA expression results of all selected studies by an expression-based meta-analysis to identify the significantly differentially expressed lncRNAs between MS patients and controls. Further, we explored their heterogeneity and expression specificity among various tissues. After that, the weighted gene co-expression network analysis (WGCNA) was performed using the expression data of lncRNAs and protein-coding genes to identify the significant modules for MS. The expression of the protein-coding genes was calculated using the same approach on lncRNA. Finally, we conducted gene set enrichment analysis (GSEA) on the co-expressed protein-coding genes in each significant module to infer the function of the differentially expressed lncRNAs potentially contributing to the pathogenesis of MS.

## Materials and Methods

### Selection of the Multiple Sclerosis-Related Ribonucleic Acid Sequencing Datasets and Studies

**Figure 1:**
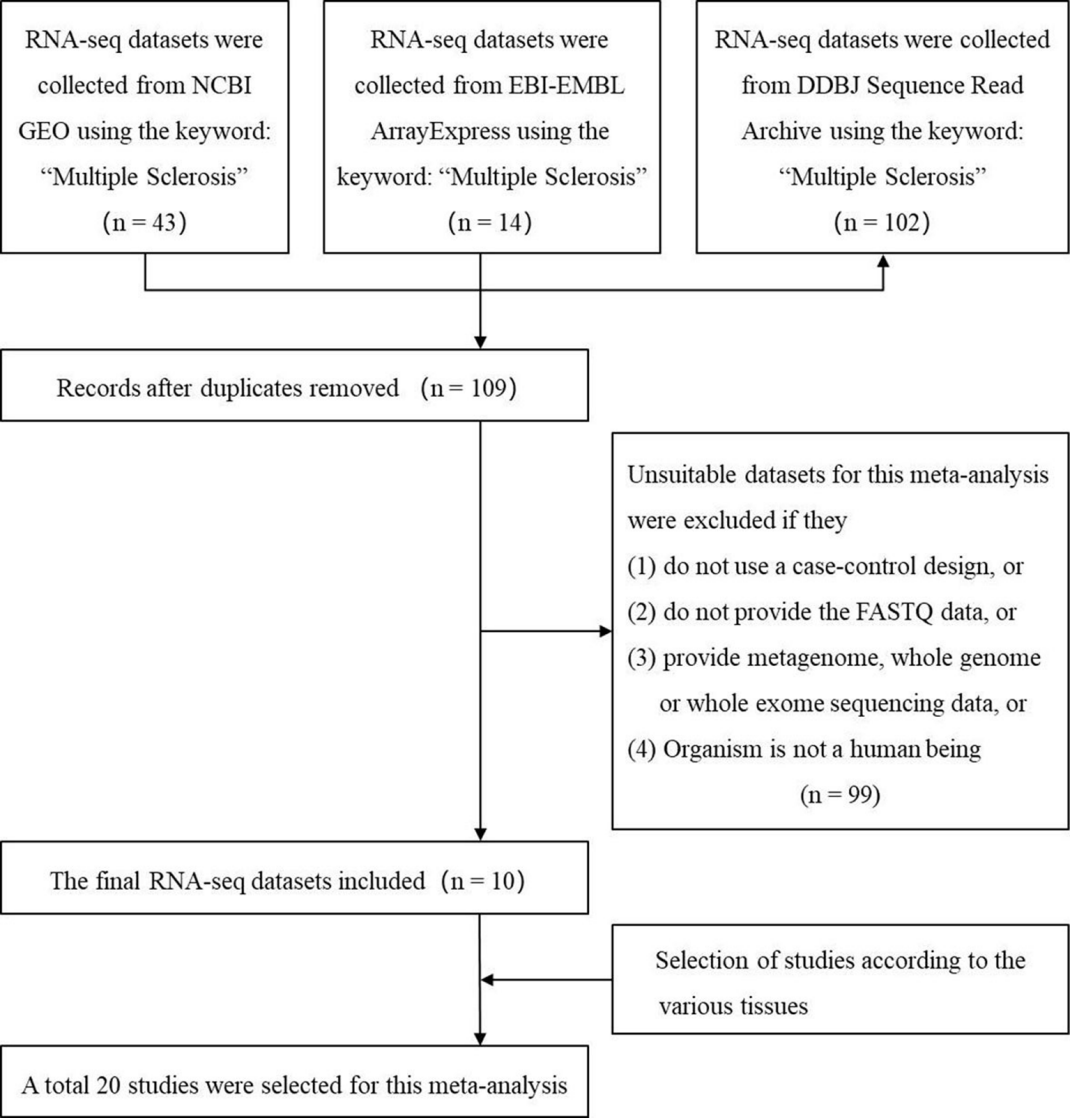
The flow chart of selecting the RNA sequencing (RNA-seq) datasets and studies which are used to identify the multiple sclerosis-related long noncoding RNAs.

We used the keyword “multiple sclerosis” to search all the possible MS-related RNA-seq datasets in three authoritative databases: GEO DataSets (Barrett et al., 2013), EBI-EMBL ArrayExpress (Athar et al., 2019), and DDBJ Sequence Read Archive (Ogasawara et al., 2013). The search was performed before the last update of the databases on May 16 2019. Then, we selected the suitable datasets using four criteria: 1) the organism in the dataset is the human being; 2) the study in the dataset is designed using the case-control method; 3) the dataset has provided the FASTQ data; (4) the FASTQ data in the dataset is not generated by metagenome, whole genome, or whole exome sequencing. Finally, the studies from these datasets based on various tissues were selected. **Figure 1** showed the workflow.

### Quantification of Long Noncoding Ribonucleic Acid Sequencing Abundance Using Ribonucleic Acid Sequencing Sequencing Data

We first downloaded the sequence data of these studies by *Prefetch* and converted them into FASTQ files using *fastq-dump* tool of the SRA Toolkit software (Leinonen et al., 2011). Next, we downloaded the reference sequences of lncRNA and protein-coding transcripts in FASTA format from NONCODE (version 5) (Fang et al., 2018) and Ensembl (release 91) (Aken et al., 2017), respectively, and further merged the two FASTA format files. Particularly, NONCODE is one of the most complete and well-annotated databases of the noncoding RNAs, and we obtained a total of 172,216 transcript sequences of 96,308 human lncRNA genes from it. Ensembl aggregated the cDNA data from National Center for Biotechnology Information (Sayers et al., 2019), UniProt (UniProt, 2015), Genome Reference Consortium (Church et al., 2011), and UCSC Genome Browser (Kent et al., 2002) databases. After removing the pseudogenes, we obtained a total of 160,040 transcript sequences of 22,810 human protein-coding genes from it. Then, we performed the quantification of the lncRNA and protein-coding transcripts simultaneously by mapping the RNA-seq reads of each study to the merged reference sequence (pseudoalignment) and calculating the count values using *Kallisto* software (Bray et al., 2016). *Kallisto* is a fast and highly accurate quantification tool for transcript abundance through k-mer lookup technique. Here, the merged reference sequences have been processed into a transcriptome index to conduct the pseudoalignment which has the same effect as the reads alignment to a given reference genome in the traditional transcript-level RNA-seq processing but can substantially reduce calculation time. For the paired-end sequencing samples, the arguments were set to defaults, i.e., the number of bootstrap samples (-b) equals 0 and the number of threads (-t) equals 1. For the single-end sequencing samples, besides these default parameter settings, we set the estimated average fragment length (-l) and the standard deviation of fragment length (-s) to 200 and 20, respectively, according to Kallisto’s recommended parameters. Finally, based on the annotation file “Transcript2Gene,” we integrated transcript-level count values of lncRNAs to calculate their corresponding gene-level count values using the R package “tximport” (Soneson et al., 2015).

### Heterogeneity Test and Meta-Analysis

To identify the significantly differentially expressed lncRNAs between MS patients and controls, we calculated and integrated the results of each study by a meta-analysis. These analyses were conducted using R package “MetaOmics,” which is a comprehensive analytical pipeline to meta-analyze multiple transcriptomic studies (Ma et al., 2019). This meta-analysis includes a normalization process same as the edgeR’s strategy and a “AW-Fisher” method to integrate data (Bullard et al., 2010; Robinson et al., 2010; Ma et al., 2019). First, we calculated the two parameters, I^2^ and P value, to measure the lcnRNA expression heterogeneity by the Cochran’s Q Statistics, which is based on a chi-square test with *k* − 1 degrees of freedom (*k* equals to the number of studies used for the meta-analysis). According to the previous studies, the heterogeneity was considered as statistically significant when I^2^ > 50% and P < 0.01 (Han et al., 2015; Li et al., 2016; Liu et al., 2017; Han et al., 2018a; Xue et al., 2018). Then, the meta-analysis was performed for each of these lncRNAs based on their count values. Particularly, the random effect model (REM) and fixed effect model (FEM) were used, respectively, for the lncRNAs with a significant heterogeneity or not. Using the REM in meta-analysis can reduce bias of the results (Kim et al., 2015; Szajewska and Kolodziej, 2015). We calculated standardized mean difference (SMD) with its 95% confidence interval (CI) to identify the differentially expressed lncRNA between the MS patients and controls (95% CI of SMD does not include zero, FDR adjusted P < 0.05). The SMD is given by the mean difference between case and control divided by the standard deviation and applies to meta-analysis when the outcome is continuous variable (e.g., expression level). Moreover, since all these samples can be split into brain and blood, we performed the meta-analysis for the two subgroups, and explored the differential expression pattern of the MS-related lncRNAs between brain and blood.

In addition, we further explored the specific target genes of the lncRNAs using LncRNA2Target v2.0 database which is authoritative source including 152,137 lncRNA-target relationships confirmed by the knockdown or overexpression analysis and binding experimental technologies, and provides web interface for searching the targets by a particular lncRNA (Cheng et al., 2019).

### Tissue Specificity Analysis of the Multiple Sclerosis-Related Long Noncoding Ribonucleic Acids

We explored the tissue expression specificity of the significantly differentially expressed lncRNAs in MS, which was important aspects of neurological disease research (usually, specifically expressed in CNS system) (Fatica and Bozzoni, 2014; He et al., 2017; Tang et al., 2019b). For this purpose, lncRNA expression data were first downloaded from the NONCODE, which were involved in primary human tissue/cell line (e.g., brain, heart, breast, lung, liver, foreskin, lung, lymph node, colon, skeletal muscle, leukocyte, HeLa cells, and fibroblasts, etc.). Then, we extracted the expression data of various tissues by the corresponding differentially expressed lncRNAs in brain, blood, and whole sample, respectively, and stored them in three independent sets. Further, based on these data, we used the Jensen-Shannon (JS) divergence, an entropy-based approach, to calculate a tissue specificity score of the differentially expressed lncRNAs according to previous study (Cabili et al., 2011). Briefly, the lncRNA expression vectors were converted to abundance density, and the distance between two tissue expression patterns was defined as the square root of JS divergence. The tissue specificity of a lncRNA expression pattern was measured through the distance between expression patterns across various tissues and predefined extreme pattern in which the lncRNA is uniquely expressed in one tissue (1 minus the distance). Thus, the metric of tissue specificity ranged from 0 to 1. The nearer the score to one, the stronger the tissue specificity becomes. Finally, using the same data, we performed the cluster analysis with Manhattan distance for differentially expressed lncRNAs in brain, blood and whole sample by R package “gplots.”

### Inferring the Functions of Multiple Sclerosis-Related Long Noncoding Ribonucleic Acids by Weighted Gene Co-Expression Network Analysis

To infer the potential biological functions of these significantly differentially expressed lncRNAs in MS, we used WGCNA approach to determine the co-expression profile of these MS-related lncRNAs and protein-coding genes, and further performed the GSEA by the co-expressed protein-coding genes. First, in the same way used for identifying MS-related lncRNAs, we quantified the abundance of the protein-coding genes and identified the significantly differentially expressed genes by a meta-analysis. Second, we constructed the co-expression network by integrating the count values of the differentially expressed lncRNAs and protein-coding genes using the R package “WGCNA” (Langfelder and Horvath, 2008). Particularly: 1) we conducted the sample clustering to check if there were any outlier samples using “hclust” function of R package “WGCNA”; 2) after quality control, we used “pickSoftThreshold” function of R package “WGCNA” to calculate the satisfactory soft threshold power β for ensuring the scale-free topology characteristics of the co-expression network; 3) based on the β value, we applied the Pearson’s method to calculate an adjacency matrix which includes the weighted correlation of all gene pairs; 4) by adjacency matrix, we used the dynamic cut-tree algorithm to construct a hierarchical clustering dendrogram and identified the co-expression modules where genes have high topological overlap with each other. Finally, we assessed the significance of the modules for MS by measuring two indices. Particularly, one of the indices is correlation between module membership (i.e., intramodular connectivity) and gene significance for MS. High correlation means that the hub genes (i.e., the genes with high connectivity in a co-expression module) of the corresponding module also tend to be highly correlated with disease states (MS or healthy) (Langfelder and Horvath, 2008). The other is the average correlation of the genes in each module with disease states. This was also applied to assess association of each module with the platforms and the tissue types, respectively.

### Pathway Analysis of the Multiple Sclerosis-Related Long Noncoding Ribonucleic Acids by Gene Set Enrichment Analysis

Based on the two indices of module significance, we selected the most significant modules of disease states to investigate the lncRNA functions in MS by GSEA. We first extracted the ID numbers of the protein-coding genes co-expressed with lncRNAs in the modules. Then, we downloaded the signaling pathway data from two common databases, Gene Ontology (GO) and Kyoto Encyclopedia of Genes and Genomes (KEGG). GO is a public resource of data on the gene functions in the biological process, molecular function, and cellular component (The Gene Ontology, 2017), and KEGG is comprehensive database which integrates the information of genes involved in signaling pathways, cellular processes, human diseases, etc. (Kanehisa et al., 2017). Finally, we used the co-expressed protein-coding genes and the signaling pathway data to conduct the GSEA of the most significant modules using R package “clusterProfiler” (Yu et al., 2012). The adjusted P value calculated by the multiple testing (Benjamini-Hochberg method) was set at less than 0.05 as the threshold of significance.

## Results and Discussion

### Results of Study Selection and Long Noncoding Ribonucleic Acid Abundance Quantification

Using keyword search and quality filtering, we identified ten MS-related RNA-seq datasets including: GSE60424, GSE66573, GSE66763, GSE89843, GSE100297, GSE120411, GSE111972, GSE123496, GSE77598, and SRP132699 from three authoritative databases. We found that the library preparation and sequencing methods in most of these datasets meet one/multiple requirements for improving the lncRNAs identification (i.e., poly-A tail selection, paired-end sequencing, and sequencing of double-stranded cDNA). Then, after the investigating the source of samples, we found that these datasets are involved in eight brain tissues (optic chiasm, corpus callosum, occipital cortex, astrocytes, frontal cortex, hippocampus, internal capsule, parietal cortex) and seven blood tissues (B cell, T cell, monocyte, platelets, neutrophils, natural killer cell, and whole blood). According to the various tissues, we selected a total of 20 studies (207 MS cases and 348 controls) for the following analysis. The detailed information of each study was shown in **Table 1**. Finally, we downloaded RNA-seq data of the samples in each study, and used them to measure lncRNA expression (count values) using *Kallisto* (Bray et al., 2016) and R package “tximport” (Soneson et al., 2015). In total, lncRNA abundance in 555 samples was quantified.

**Table 1:** Summary of the 20 selected studies for the meta-analysis. NK, natural killer cell.

Study Number	Dataset	Tissue	Year	No. of cases	No. of controls	Sequencing platform	RNA-seq library type		
							Poly-A tail select	Sequencing of double-stranded cDNA	Read type
1	GSE60424	B-cells	2014	6	4	Illumina HiScanSQ	Yes	Not described	Paired-end
2	GSE60424	Monocytes	2014	6	4	Illumina HiScanSQ	Yes	Not described	Paired-end
3	GSE60424	Neutrophils	2014	6	4	Illumina HiScanSQ	Yes	Not described	Paired-end
4	GSE60424	NK	2014	3	4	Illumina HiScanSQ	Yes	Not described	Paired-end
5	GSE60424	T-cells	2014	12	8	Illumina HiScanSQ	Yes	Not described	Paired-end
6	GSE60424	Whole blood	2014	6	4	Illumina HiScanSQ	Yes	Not described	Paired-end
7	GSE66573	Whole blood	2015	6	8	Illumina HiSeq 2500	Yes	Yes	Paired-end
8	GSE66763	T-cells	2015	10	6	Illumina HiSeq 2500	Not described	Not described	Paired-end
9	GSE77598	Monocytes	2016	5	3	Illumina HiSeq 2000	Not described	Not described	Paired-end
10	GSE89843	Platelets	2017	58	234	Illumina HiSeq 2500	Yes	Yes	Single-end
11	GSE100297	Optic chiasm	2017	5	5	Illumina HiSeq 3000	Yes	Yes	Single-end
12	GSE111972	Corpus callosum	2018	10	11	Illumina NextSeq 500	Yes	Not described	Single-end
13	GSE111972	Occipital cortex	2018	5	5	Illumina NextSeq 500	Yes	Not described	Single-end
14	GSE120411	Astrocytes	2018	24	18	Illumina HiSeq 2500	Yes	Not described	Single-end
15	SRP132699	Monocytes	2018	20	5	Illumina HiSeq 2500	Not described	Not described	Single-end
16	GSE123496	Corpus callosum	2019	5	5	Illumina HiSeq 3000	Yes	Yes	Single-end
17	GSE123496	Frontal cortex	2019	5	5	Illumina HiSeq 3000	Yes	Yes	Single-end
18	GSE123496	Hippocampus	2019	5	5	Illumina HiSeq 3000	Yes	Yes	Single-end
19	GSE123496	Internal capsule	2019	5	5	Illumina HiSeq 3000	Yes	Yes	Single-end
20	GSE123496	Parietal cortex	2019	5	5	Illumina HiSeq 3000	Yes	Yes	Single-end

### Heterogeneity Test and Meta-Analysis

**Figure 2:**
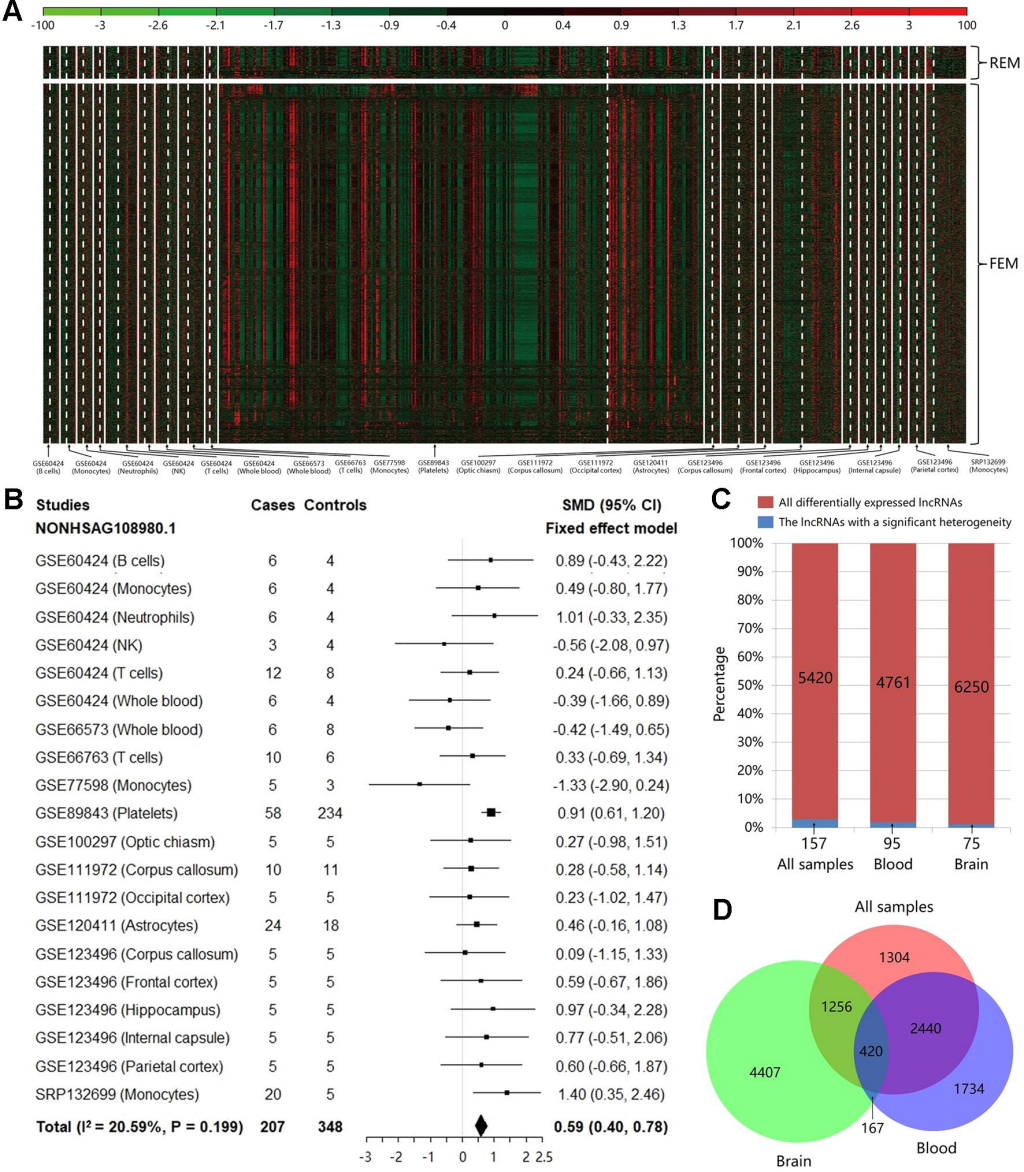
The results of heterogeneity test and meta-analysis for all samples and subgroups. **(A)** The expression level of the significantly differentially expressed long noncoding RNAs (lncRNAs) in each study after meta-analysis. The random effect model was used for 157 lncRNAs with a significant heterogeneity, while the fixed effect model was used for 5,263 non-heterogeneous lncRNAs. The details can be clearly viewed by enlarging the electronic version. **(B)** The forest plot for the meta-analysis of the lncRNA NONHSAG108980.1 which is the most significant result associated with an increased risk of MS (SMD = 0.59, 95% CI = 0.40−0.78, P = 1.89×10^−9^). **(C)** The bar plot showing the results of heterogeneity test in each group. For all samples, the proportion of lncRNAs with a significant heterogeneity is not high (about 2.90%), and this percentage is further decreased to about 1.99 and 1.20% in blood and brain, respectively. **(D)** The Venn diagram exhibiting the overlap among the significantly differentially expressed lncRNAs that are identified using brain tissues, blood tissues, and all samples.

Based on the count values of the 96,308 lncRNAs in 20 studies, the meta-analysis was performed to calculate SMD value with its 95% CI for each lncRNA using REM/FEM. Heterogeneity test showed that only about 2.90% lncRNAs have the significant heterogeneity (I^2^ > 50% and P < 0.01). Therefore, the homogeneous unbiased results could be identified in >97% lncRNAs by FEM. For the remaining lncRNAs of significant heterogeneity, REM could reduce resulting bias. In total, 5,420 lncRNAs were identified significantly differentially expressed between MS cases and controls, which included 368 downregulated and 5,052 upregulated lncRNAs (shown in **Figure 2A** and **Supplementary Table S1**). For example, the **Figure 2B** exhibited the meta-analysis results of the lncRNA NONHSAG108980.1 which shows the most significant association with an increased risk of MS (SMD = 0.59, 95% CI = 0.40−0.78, P = 1.89×10^−9^). Then, to investigate the heterogeneity of the lncRNA expression profile in various tissues, we split the samples into brain and blood tissue, and performed the heterogeneity test and meta-analysis for subgroups. We found that not only the proportion of lncRNAs with a significant heterogeneity was not high for the whole samples, but also this percentage is further reduced to about 1.99 and 1.20% in blood and brain, respectively (**Figure 2C**). Finally, we explored the difference of the differentially expressed lncRNAs identified in various tissues. We found that there was the higher specificity for these lncRNAs identified in brain compared with them identified in blood. Particularly, about 60.06% of the 5,420 differentially expressed lncRNAs can also be identified in the blood, while percentage is only 26.82% in brain (**Figure 2D**). Moreover, the total number of upregulated lncRNAs is far more than that of the downregulated ones in the blood (**Supplementary Table S2**) and the brain (**Supplementary Table S3**), which indicated that MS risk was related to lncRNA overexpression.

In addition, previous studies found that lncRNAs were modestly evolutionarily conserved in sequence (Guttman et al., 2009; Iyer et al., 2015). Therefore, we explored the conservation in sequence of these differentially expressed lncRNAs using conservation constrain search in NONCODE which contains the conservation information of lncRNAs in 13 common model organisms (i.e., human, chimp, gorilla, orangutan, rhesus, mouse, rat, cow, pig, opossum, platypus, chicken, and zebrafish). The results showed that only 0.11% of the differential lncRNAs were conserved in sequence among all these 13 organisms, while this percentage is increased to 28.5% in primates (human, chimp, gorilla, orangutan, and rhesus).

### Tissue Specificity Analysis of the Multiple Sclerosis-Related Long Noncoding Ribonucleic Acids

**Figure 3:**
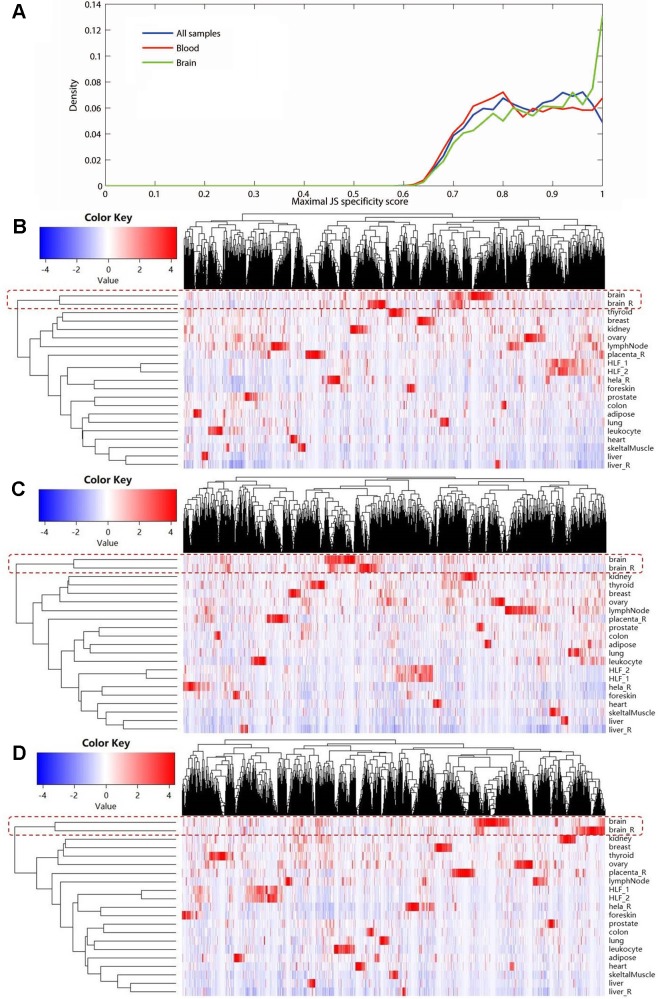
The tissue specificity of the multiple sclerosis-related long noncoding RNAs (lncRNAs) based on expression data from NONCODE database. **(A)** Tissue specific expression measured by Jensen-Shannon divergence. The distributions of the maximal tissue specificity scores showed the high tissue specificity of the differentially expressed lncRNAs identified using whole (blue), brain (green), and blood sample (red), respectively. The **(B)** to **(D)** showed the hierarchical clustering heatmap for expression of these lncRNAs in primary human tissues and cell lines. These differentially expressed lncRNAs identified using whole **(B)**, brain **(C)**, and blood sample **(D)** are all highly specifically expressed in brain tissue. The Manhattan distance was used to perform all of the three cluster analyses.

Using expression data of NONCODE database, we performed the JS divergence metric and the cluster analysis to explore the tissue specificity of MS-related lncRNAs. The results of JS divergence metric showed that the MS-related lncRNA had high tissue specificity when used the brain, blood and whole samples (**Figure 3A**). For cluster analysis, relied on the same data, we further compared the expression patterns of these differentially expressed lncRNAs in various human tissues and cell lines. We found that the differentially expressed lncRNAs identified based on whole sample were highly specifically expressed in brain tissue (**Figure 3B**). Similarly, we observed a significant brain-specific expression for the differentially expressed lncRNAs identified based on brain sample (**Figure 3C**). Interestingly, although the differentially expressed lncRNAs were identified from blood sample, their expressions were still highly specific in brain tissue (**Figure 3D**). These results are consistent with the findings of the previous step and our recently published study (Han et al., 2019), which suggest that MS possesses the characteristics of the CNS disorder in lncRNA dysregulation.

### Inferring the Functions of Multiple Sclerosis-Related Long Noncoding Ribonucleic Acids by Weighted Gene Co-Expression Network Analysis

**Figure 4:**
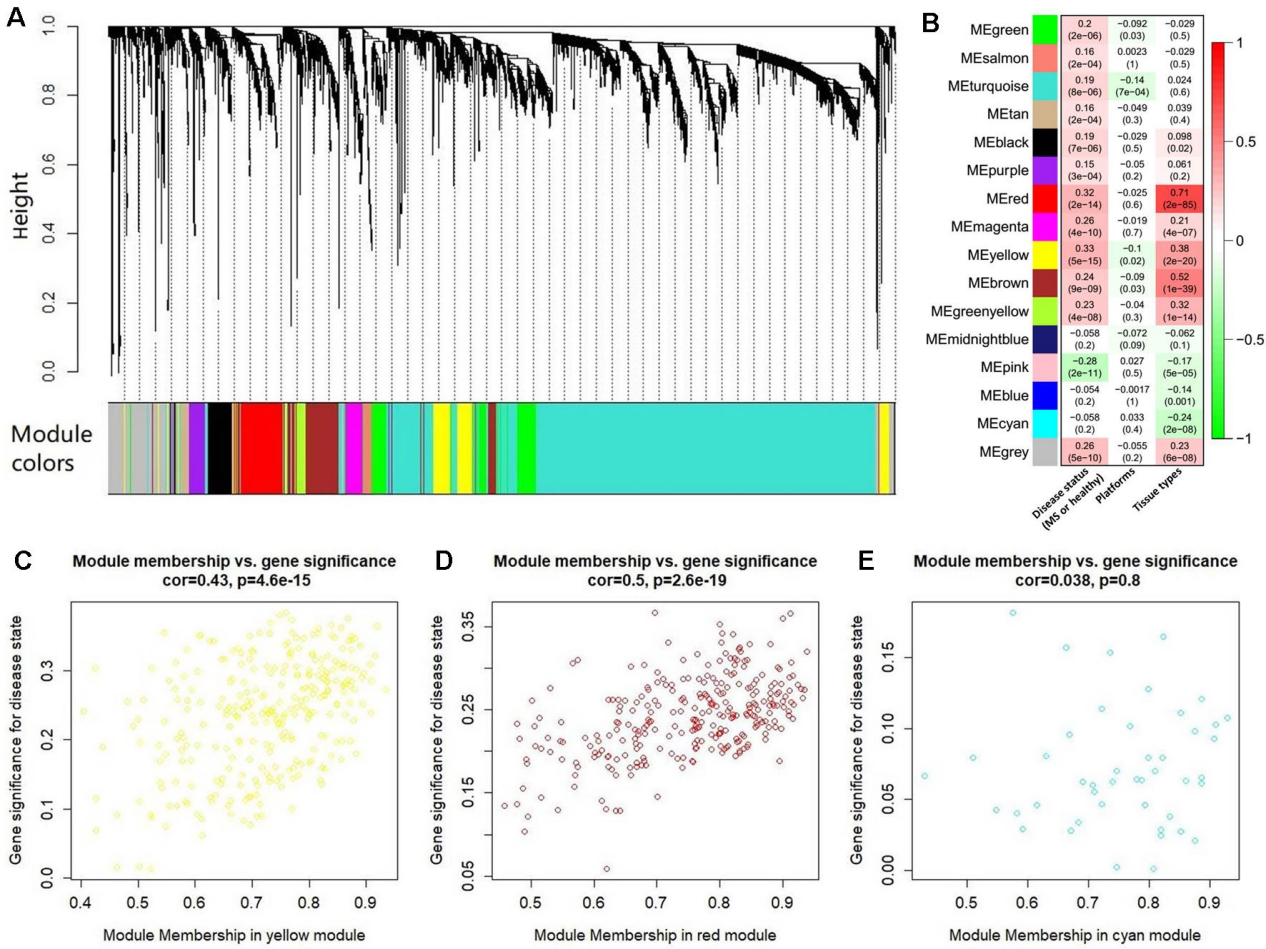
The co-expression network analysis of the differentially expressed long noncoding RNAs (lncRNAs) and protein-coding genes. **(A)** The clustering dendrogram of these co-expressed lncRNAs and protein-coding genes. There are 15 clustered modules in the hierarchical clustering dendrogram which is constructed by a dynamic cut-tree algorithm. These clustered modules are marked as 15 different colors, respectively, i.e., yellow, turquoise, tan, salmon, red, purple, pink, midnight blue, magenta, green yellow, green, cyan, brown, blue, and black. **(B)** The heatmap for the association of each module with the disease states, platforms, and tissue types. Each cell represents a module, and contains the correlation r and corresponding P value (in brackets). Panels **(C)** to **(E)** show the results of correlation between the module membership and the gene significance in MEyellow, MEred, and MEcyan, respectively. The results of other modules were described in **Supplementary Figure S3**.

After abundance quantification together with meta-analysis, we identified 2,051 protein-coding genes significantly differentially expressed between MS patients and controls (**Supplementary Table S4**). Then, we combined the count values of 2,051 differentially expressed protein-coding genes and 5,420 MS-related lncRNAs to perform the WGCNA. By quality control, we removed three outlier samples whose minimum cluster size less than 5 and cutting height less than 4.0×10^6^ (**Supplementary Figure S1**). The satisfactory soft threshold power β was set as 9 when the model fitting index R^2^ equals 0.8 and the mean connectivity is close to 0 simultaneously (**Supplementary Figure S2**). Finally, we constructed a co-expression network which includes 1,938 protein-coding genes and 5,022 lncRNAs, and according to the interconnectedness of gene pairs, they were clustered into 15 modules in network (MEyellow, MEturquoise, MEblue, MEsalmon, MEred, MEpurple, MEpink, MEmagenta, MEgreen, MEmidnightblue, MEcyan, MEtan, MEgreenyellow, MEbrown, and MEblack) (**Figure 4A**). Moreover, to assess the significance of these modules for MS, we calculated two types of correlations as the index. The results of the average correlation of the genes in each module with the disease states showed that MEyellow is the most associated module with MS (r = 0.33, P = 5×10^−15^), and the following three are MEred (r = 0.32, P = 2×10^−14^), MEpink (r = −0.28, P = 2×10^−11^), and MEbrown (r = 0.24, P = 9×10^−9^). This was also applied to assess the association of each module with the platforms and the tissue types, respectively. Consistently, we found that the MEred (r = 0.71, P = 2×10^−85^), MEbrown (r = 0.52, P = 1×10^−39^), and MEyellow (r = 0.38, P = 2×10^−20^) were most significantly associated with the tissue types. While there is no module strongly associated with platforms (**Figure 4B**). These findings are generally consistent with the result of the correlation between the module membership and the gene significance for MS. For example, MEyellow and MEred are the top two module with the high average correlation of genes with disease states, and they also show a high correlation between module membership and gene significance (cor = 0.43, P = 4.6×10^−15^ and cor = 0.50, P = 2.6×10^−19^, respectively) (**Figures 4C, D**). On the contrary, MEcyan shows a very low level both for the two types of correlations (r = −0.058, P = 0.2 and cor = 0.038, P = 0.8) (**Figure 4E**).

In addition, we also perform a WGCNA with the satisfactory soft threshold power β = 9 using all the quantified genes. We found that these genes are clustered into 119 modules in the network, and about 82.2% differentially expressed genes are clustered into 16 of the 119 modules (including a gray one). We also found that these modules show low/modest association with MS (the correlation coefficients are < 0.19). These results reflect the similar distribution of the differentially expressed genes between using all and filtering genes in this WGCNA, and imply that the extra genes may mask the association of the differentially expressed genes with MS.

### Pathway Analysis of the Multiple Sclerosis-Related Long Noncoding Ribonucleic Acids by Gene Set Enrichment Analysis

**Figure 5:**
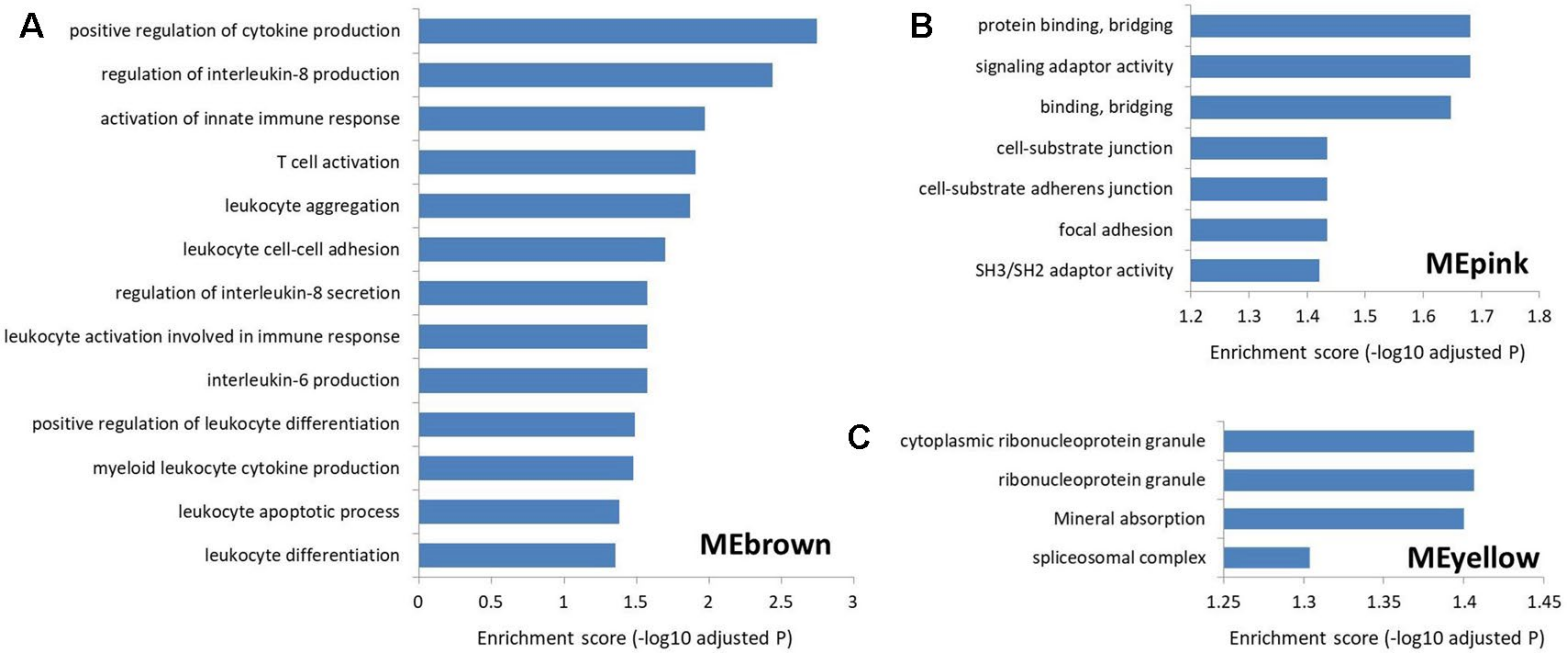
The Gene Ontology and Kyoto Encyclopedia of Genes and Genomes pathway enrichment in the three most significant modules for multiple sclerosis. **(A)** The enrichment for MEbrown. The protein-coding genes co-expressed with the MS-related lncRNAs in this module are mainly involved in leukocyte and interleukin-related immune response. **(B)** The enrichment for MEpink. The co-expressed protein-coding genes in this module are mainly associated with the intercellular junction and signaling transmission. **(C)** The enrichment for MEyellow. The co-expressed protein-coding genes in this module are mainly related to the ribonucleoprotein.

To explore the function of lncRNAs in MS, we performed GSEA in the four most significant modules for MS based on the two types of correlations, i.e., MEyellow (r = 0.33, P = 5×10^−15^ and cor = 0.43, P = 4.6×10^−15^), MEred (r = 0.32, P = 2×10^−14^ and cor = 0.50, P = 2.6×10^−19^), MEpink (r = −0.28, P = 2×10^−11^ and cor = 0.63, P = 3.5×10^−14^), and MEbrown (r = 0.24, P = 9×10^−9^ and cor = 0.32, P = 4.7×10^−9^). We found no significantly enriched pathway related to the MEred. Based on the result of LncRNA2Target, we identified that two differentially expressed lncRNAs in MEred could target the MS-related genes. Particularly, two target genes (CDH1 and CDH2) of the lncRNA NONHSAG081583.2 encoded cadherin protein which is the most abundant adhesion molecules participating in nerve conduction in synaptic junctions and the proinflammatory cytokines in MS can downregulate its expression (Minagar et al., 2003; Tian et al., 2009). The lncRNA NONHSAG000840.2 targets a MS-related gene NOTCH2, and reducing NOTCH2 in the proinflammatory monocytes can increase the frequency of the nonclassical monocytes and neutralizing antidrug antibody induction in IFN-β treated MS patients (Adriani et al., 2018). For MEbrown, the co-expressed protein-coding genes were mainly involved in leukocytes and interleukin-related immune response (**Figure 5A**and **Supplementary Table S5**), which was similar to the finding of our recent study (Han et al., 2019). Many genomic variants in the human leukocyte antigen complexes and interleukin receptor were identified significantly associated with susceptibility of MS (Rubio et al., 2002; Teutsch et al., 2003; Lundmark et al., 2007; Hollenbach and Oksenberg, 2015; Tang et al., 2019a). The protein-coding genes in MEpink are mainly associated with intercellular junction and signaling transmission (**Figure 5B**). Previous studies found that the defect of axon-glial signaling transmission caused by the oligodendrocyte gap junction loss and disconnection contributes to MS pathogenesis (Brand-Schieber et al., 2005; Markoullis et al., 2012; Markoullis et al., 2014). The results of LncRNA2Target showed that lncRNA NONHSAG049754.2 in MEyellow targets the MS-related gene TNFRSF10A. This gene encodes the receptor of tumor necrosis factor (TNF) cytokines which plays a important role in inflammation regulations and is related to susceptibility of developing MS (De-la-Torre et al., 2019). The protein-coding genes in the MEyellow are related to ribonucleoprotein (**Figure 5C**). Ribonucleoprotein is a kind of ribonucleic acid-binding protein which participates in the mRNA splicing (Guthrie, 1991). Previous study showed that as an important autoantigen in the neuroimmune disease, the ribonucleoprotein significantly more often interact with the autoantibodies in MS cerebrospinal fluids compared with controls (Sueoka et al., 2004; Yukitake et al., 2008). The following studies further identified a ribonucleoprotein-related lncRNA, TNF-α, and heterogeneous nuclear ribonucleoprotein L, which was significantly upregulated and produced transcriptional activating complexes to promote TNF-α expression by cooperating with ribonucleoprotein in the circulating blood cells of MS (Li et al., 2014; Eftekharian et al., 2017). Given that MEyellow is the most significant module for MS, we inferred that one of the key mechanisms of lncRNAs in MS is associated with the regulation of ribonucleoprotein and TNF cytokines receptor.

## Conclusions

In this study, we comprehensively collected MS-related RNA-seq data from a variety of studies, and integrated these data by an expression-based meta-analysis to assess the affection of lncRNAs on the MS pathogenesis on genome scale. We identified a total of 5,420 lncRNAs significantly differentially expressed between MS patients and controls. Then, the subgroup analysis found a small heterogeneity of the lncRNA expression profile between the brain and blood tissues. Further, the specificity analysis of multiple tissues showed that the differentially expressed lncRNAs (including identified using brain, blood, and whole sample) are highly specifically expressed in brain tissue. Finally, the result of GSEA and WGCNA demonstrated that the potential important function of lncRNAs in MS may be involved in the regulation of ribonucleoprotein and TNF cytokines receptor. All in all, we performed a strategy to resolve the inconsistent MS-related lncRNA findings in previous studies, and explore the functions of these lncRNAs in MS. The findings of this study will be benefit to improve the understanding of the pathogenesis of MS.

## Data Availability Statement

Publicly available datasets were analyzed in this study. This data can be found here: https://www.ncbi.nlm.nih.gov/gds, https://www.ebi.ac.uk/arrayexpress, https://ddbj.nig.ac.jp/DRASearch.

## Author Contributions

ZH and FZ designed the research. ZH, FZ, JH, and WX collected the data. ZH performed the research, analyzed data, and wrote the paper. FZ reviewed and modified the manuscript. All authors discussed the results, and contributed to the final manuscript. All authors read and approved the final manuscript.

## Funding

This work was funded by National Key R&D Program of China (2018YFC0910500), National Natural Science Foundation of China (81872798), Fundamental Research Fund for Central Universities (10611CDJXZ238826, 2018QNA7023, 2018CDQYSG0007 & CDJZR14468801), and Innovation Project on Industrial Generic Key Technologies of Chongqing (cstc2015zdcy-ztzx120003).

## Conflict of Interest

The authors declare that the research was conducted in the absence of any commercial or financial relationships that could be construed as a potential conflict of interest.
