# Supplementary material for: Integrating the Ribonucleic Acid Sequencing Data From Various Studies for Exploring the Multiple Sclerosis-Related Long Noncoding Ribonucleic Acids and Their Functions
Source: Front Genet. 2019 Nov 12;10:1136. doi: 10.3389/fgene.2019.01136 (PMC6861379; doi:10.3389/fgene.2019.01136)
Supplement: Supplementary file 6 [file Presentation_1.pdf]

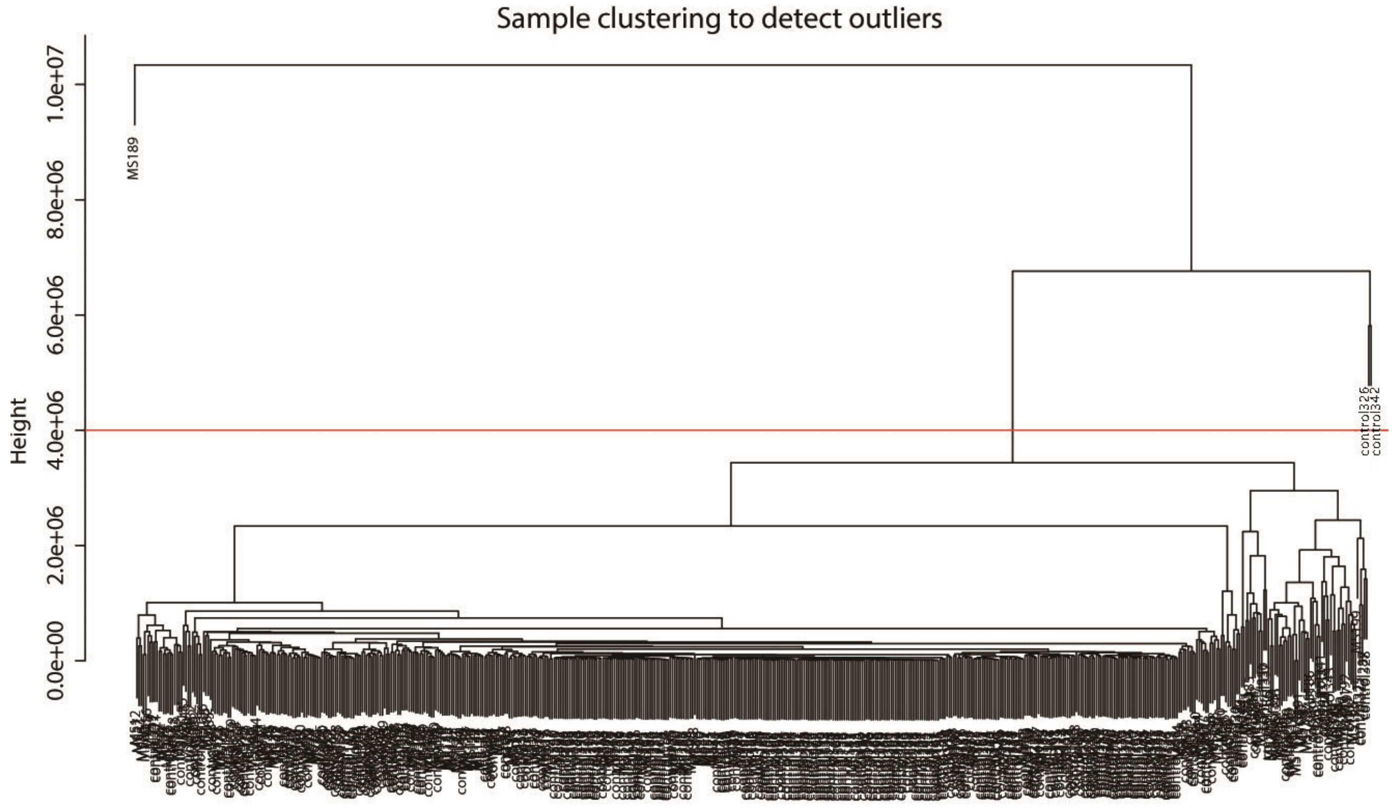

Figure S1 Sample clustering to detect the outliers. The sample clustering is based on the expression profiles of the significantly differentially expressed lncRNAs and protein-coding genes in each individual. It shows that sample MS189, control326 and control342 are three obvious outliers according to the criterion (minimum cluster size = 5 and cutting height = 4.0×10^6), and therefore they are removed for the following analysis.

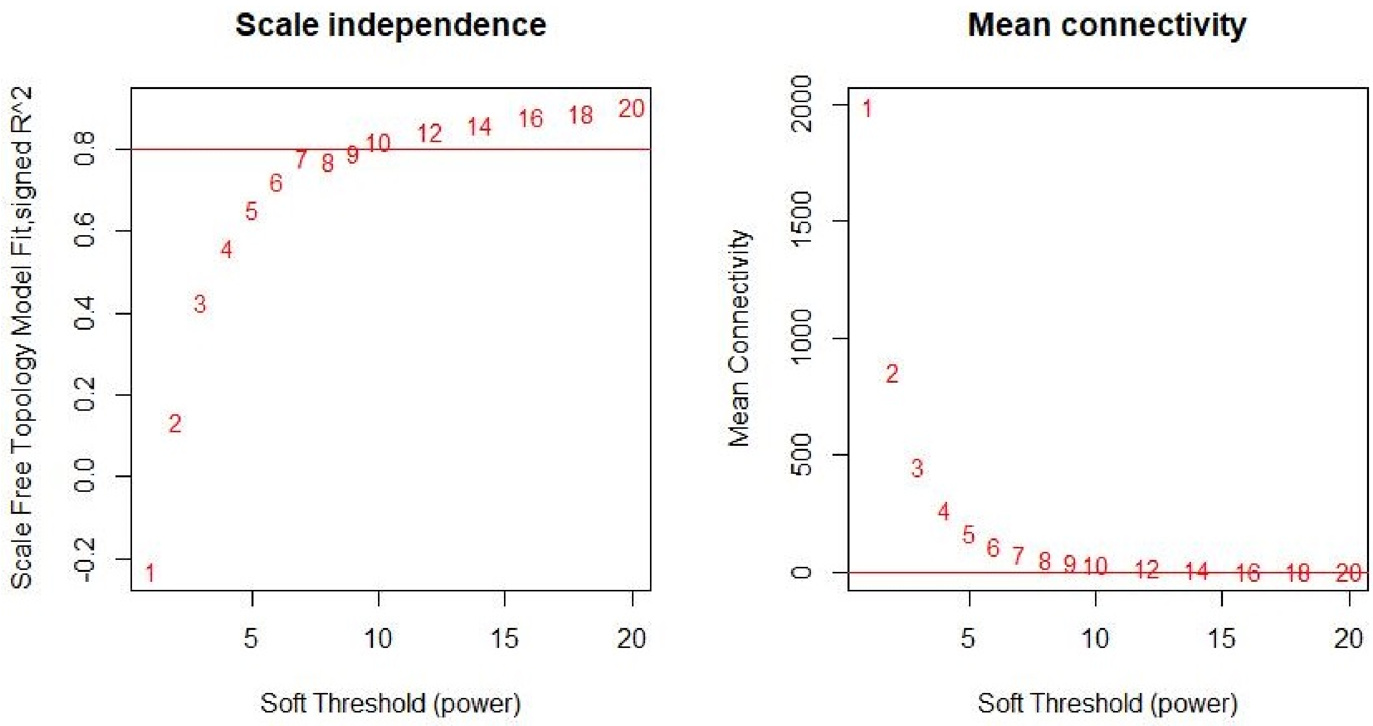

Figure S2 Determination of satisfactory soft threshold power by network topology analysis. In the left panel, the soft threshold power equals 9 when the model fitting index R-squared reaches 0.8 for the first time. In the right panel, the soft threshold power still equals 9 when the mean connectivity approaches 0. Therefore, the optimum power value is set at 9 to ensure that the co-expression network follows scale-free topology criterion.

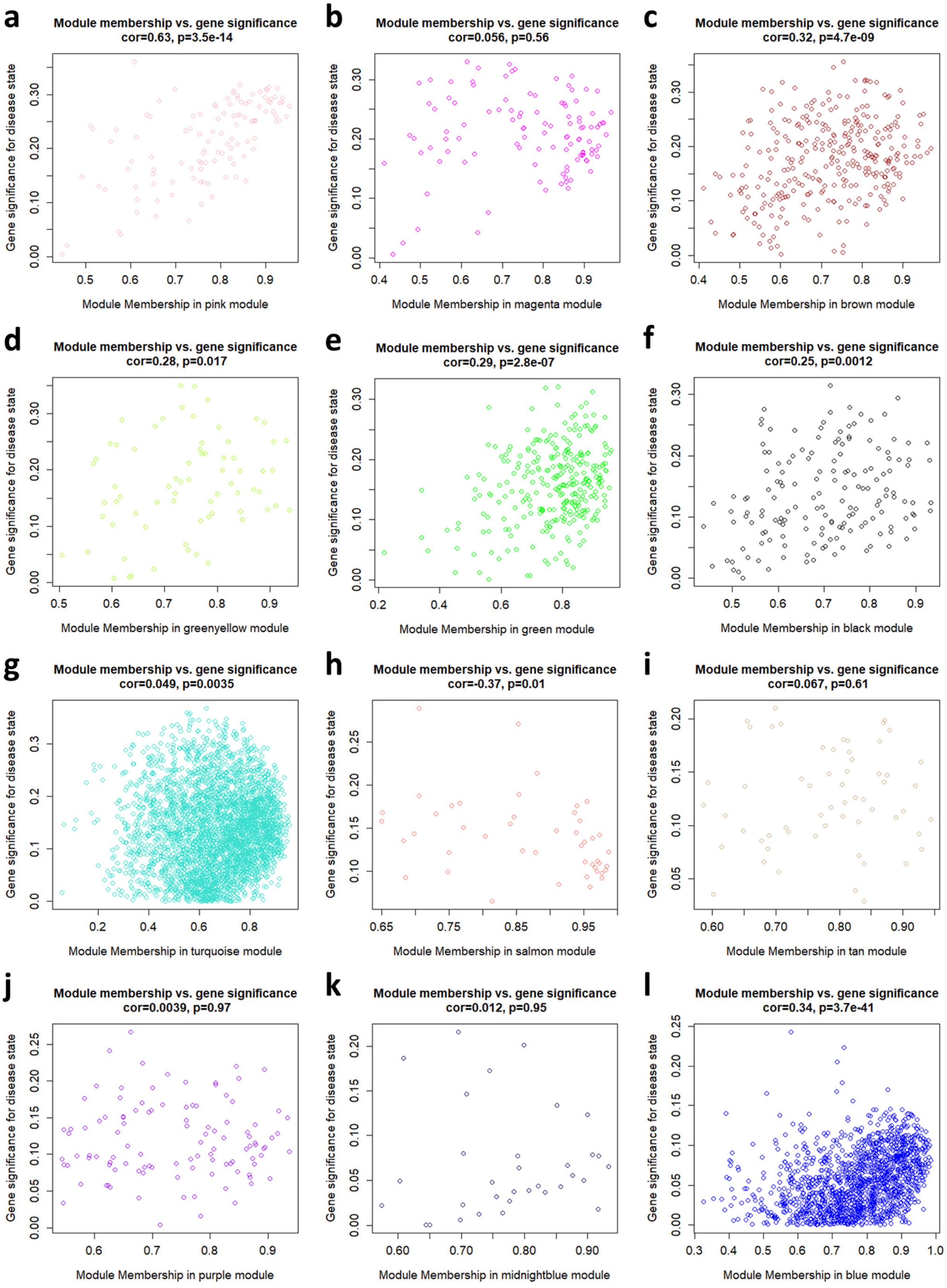

Figure S3 The plot of module membership vs. gene significance for each module. From MEpink (**a**) to MEblue (**l**), the average correlation of the genes in each module with the disease states weakens successively, which is consistent with the correlation between the module membership and the gene significance in most of these modules.
